# Experiences of Fundamental Care Needs After Cytoreductive Surgery Combined With Hyperthermic Intraperitoneal Chemotherapy: A Qualitative Study

**DOI:** 10.1155/nrp/6689299

**Published:** 2026-05-16

**Authors:** Pernilla Bangsparr, Jenny Jakobsson

**Affiliations:** ^1^ Department of Surgery and Gastroenterology, Skåne University Hospital, Inga Marie Nilssons Gata 47, Malmö, 20502, Sweden, skane.se; ^2^ Faculty of Health and Society, Institution of Care Science, Malmö University, Jan Waldenströms Gata 25, Malmö, 20502, Sweden, mah.se

## Abstract

**Aim:**

To explore how patients experience their fundamental care needs during the early phase of recovery after cytoreductive surgery combined with hyperthermic intraperitoneal chemotherapy.

**Design:**

Qualitative with deductive approach.

**Methods:**

Thirteen participants were interviewed approximately 2 weeks after surgery. A deductive, qualitative content analysis was performed based on the Fundamentals of Care framework.

**Results:**

All three dimensions in the Fundamentals of Care framework were represented, most of which were physical needs: personal hygiene, toilet needs, nutrition, mobility, sleep and rest, and physical well‐being. The most evident psychological needs were participation and information. Participants highly valued the nurse–patient partnership as promoting recovery but expressed that environmental factors could hamper care and recovery.

**Conclusion:**

Physical needs are commonly interrelated with psychological needs and the relation with nurses, highlighting the complexity of nursing and importance of a person‐centred approach. Environmental factors and organisational policies impact patients’ opportunities to have their physical and psychological needs met.

**Summary**
• Implications
◦ Structured, person‐centred care based on patients’ abilities, resources and fundamental needs is warranted to provide high‐quality care. Hence, nurses should focus on the partnerships with patients and, if possible, relatives to create mutual goals that are repeatedly evaluated.
• Impact
◦ Patients recovering from cytoreductive surgery (CRS) combined with hyperthermic intraperitoneal chemotherapy (HIPEC) form an understudied group with a challenging and protracted recovery. The study can guide nurses performing postoperative care to acknowledge patients’ fundamental care needs and provide insights into hospital organisations about the influence of care environment and policies on recovery.
• Patient or public contribution
◦ No patient or public contribution.

## 1. Introduction

Colorectal cancer is the third most common cancer diagnosis worldwide, with approximately 1.9 million new cases every year [1]. About 8%–13% of persons with colorectal cancer are affected by peritoneal surface malignancy (PSM) with tumour dissemination at the peritoneal surface or visceral organs [2]. The spreading becomes uncontrolled as tumour cells circulate within the peritoneal fluid. Tumour cell implantation on visceral organs, intestinal mesentery and peritoneal surfaces can lead to bowel obstruction, malnutrition and death [3]. The most common form of PSM arises from other types of gastrointestinal cancer, such as colorectal cancer, but PSM can also come from the peritoneum itself [4]. Without an aggressive, multimodal approach, PSM is associated with a poor prognosis, with a median survival of only 5 months [2, 5]. However, since the 1990s, CRS combined with HIPEC has become a treatment standard for many PSMs and is increasingly used as a curative treatment [5–7]. Five‐year survival rates after CRS combined with HIPEC range from 23% to 52%, and patients with limited PSM have the highest chance of survival [2, 8]. CRS combined with HIPEC can also be performed on more unusual forms of malignancies, such as peritoneal mesothelioma (i.e., cancer that arises from the peritoneum) and pseudomyxoma peritonei, which is a mucinous tumour of the appendix that spreads into the peritoneal cavity in the form of gelatinous ascites [6, 8].

The first step in the CRS procedure is to remove the primary tumour, if not done previously, followed by an extensive peritoneal stripping of tumour nodules from the surfaces inside the abdominal cavity [9]. After the CRS, the HIPEC procedure is performed to treat micro‐metastases on the peritoneal surface using locally administered chemotherapy that is heated to 42°C–43°C. The effect is the result of the combination of the locally administered chemotherapy and the direct toxicity of hyperthermia. The HIPEC procedure lasts 30–90 min [10].

All surgical procedures are followed by a period of recovery starting when anaesthesia is discontinued and lasting until the patient reaches preoperative levels of well‐being and health [11]. The CRS combined with HIPEC procedure is a complex intervention associated with high risk for morbidity and protracted recovery [2]. This has been shown, for example, in a prospective study by Dodson et al., reporting that CRS combined with HIPEC was associated with morbidity and temporary impairments in quality of life (QoL), even though most patients recovered to a satisfactory overall QoL within 6 months or earlier. Furthermore, it was shown that QoL at baseline was associated with postoperative morbidity, mortality and survival outcomes [12]. Another prospective study showed that patients’ QoL related to postoperative symptoms such as pain, appetite loss and constipation did not return to preoperative levels until after 6 months. Symptoms such as fatigue, diarrhoea, dyspnoea and sleep disturbance were not recovered until after 12 months [13]. Similar results were shown in a systematic review by Leimkühler et al. reporting that fatigue, dyspnoea, diarrhoea and sleep disturbance were the most common symptoms in the first 6 months after surgery. After 12 months, only diarrhoea was described as affecting patients’ QoL [14].

The earliest recovery period, when patients are still hospitalised, was described in an interview study by Eriksson et al. [15]. The patients in the study experienced an emotional roller coaster, in which thoughts of death were mixed with hallucinations and nightmares. Physical symptoms caused by the treatment were described as overwhelming and included physical and mental fatigue, weakness, difficulties in sleeping, pain, nausea and loss of appetite. However, recovery during hospitalisation was experienced as facilitated by continuous information, being allowed to participate in their own care and receiving support from the healthcare professionals, with opportunities for small chats. In another study by Leo Swenne et al. [16], patients were interviewed 2–6 months after surgery. This study described how patients had experienced pain in their abdominal muscles, poor appetite, diarrhoea, fatigue and sleeping difficulties during the first 6 months of recovery. The patients were interviewed again after 14 months [17]. In those interviews, they described a shift of focus to finding their new self and adapting to new postsurgery circumstances. However, the patients still felt mental fatigue and were worried about the return of disease and the lack of a rehabilitation plan. In another prospective, interview study performed at 3, and 6–12 months after surgery, Francescutti et al. [18] reported that the patients remembered pain and pain management as the largest problem during their hospital stay. Later, after being discharged from the hospital, the patients felt depressed because they did not have the energy to be as active as they used to be. However, life was described as gradually returning to normal, for some as early as 4 months after treatment. For others, it took longer than 6 months. Although life sooner or later was experienced as normal, the participants described having persistent problems with sleep, pain and altered bowel activity that required dietary adjustments [18].

As most previous studies have a prospective and longitudinal design [16–18], little is known about how patients experience their recovery during the earliest phase after surgery (i.e., during hospital stay) and what factors they perceive as having an impact on recovery. To more thoroughly describe patients’ experiences of recovering from CRS combined with HIPEC, only a few qualitative studies have been conducted. Therefore, the aim of this study was to explore how patients experience their fundamental care needs during the early phase of recovery after CRS combined with HIPEC.

## 2. Methods

### 2.1. Design

The study employed a descriptive, narrative design. Data were collected through semistructured interviews and analysed using deductive qualitative content analysis as described by Elo and Kyngäs [19, 20]. The Fundamentals of Care framework (FoC) was used as a guide during analysis.

### 2.2. Theoretical Framework

The FoC framework is described by Feo et al. [21] as highlighting certain fundamental aspects of nursing. Muntlin and Jangland [22] have translated and interpreted the framework to be implemented into Swedish healthcare, where it is increasingly emphasised not only within healthcare but also in nurse education. Nursing interventions can be based on the framework to give the patients good quality care. The FoC framework covers the following three dimensions [22]:

- Integrating the physical, psychosocial and relational aspects of nursing
- Establishing a care partnership between nurse and patient/relatives
- Considering the context in which care is provided

The partnership between the nurse and the patient is a central part of the FoC framework. Being present and having knowledge about the patients’ health are basic skills for understanding the patients’ fundamental care needs. This involves seeing the patients as experts on their experiences when a partnership is created, and relatives can advantageously be included. Working in a partnership involves creating goals together with the patients and following up to see that the goals are reached. Acknowledging patients’ physical and psychological needs is an element of caring that is essential and unique for every patient. However, care is given in different contexts, and as a result, the prerequisites to provide care can be affected by both environmental and organisational factors [22, 23].

### 2.3. Study Setting and Recruitment

This study was performed in Sweden, where approximately 150–200 patients annually undergo CRS combined with HIPEC [24]. At the time of data collection, patients at the study site were hospitalised for approximately 2–3 weeks depending on how well their recovery progressed.

Patients were consecutively recruited to the study throughout 1 year (October 2022 to October 2023) from one of the four university hospitals in Sweden that perform CRS with HIPEC. Recruitment lasted until not further information was added during interviews. To be eligible, patients must have been scheduled to undergo CRS with HIPEC. Written information about the study was given to all eligible patients during their routine preoperative visit at the surgical outpatient clinic about 1 week before surgery. The information was distributed by the contact nurses who met the patients during the visits. Patients were admitted to the surgical ward the day before surgery, and then, the first author contacted them to give additional information orally and create the opportunity to ask questions about the study before deciding whether to participate or not. If the patients were interested in participating, a preliminary day and time for when the interview would take place was decided together.

### 2.4. Inclusion and Exclusion Criteria

Inclusion criteria for the study were adult patients (> 18 years) who had completed CRS combined with HIPEC due to colorectal cancer, peritoneal mesothelioma or pseudomyxoma peritonei. Further inclusion criteria were an expected ability to participate in an interview approximately 2 weeks after surgery. Exclusion criteria were patients with insufficient knowledge of the Swedish language and patients with impaired cognitive or verbal ability.

### 2.5. Data Collection

A total of 18 patients met the inclusion criteria during the recruitment period and were invited for participation. Four patients declined to participate in the study. Hence, a total of 14 interviews were performed. Of those, one interview was excluded because of technical error during audio recording. Data collection was performed through individual, semistructured interviews approximately 2 weeks after surgery and treatment. This time point was chosen because previous studies suggest that the first 2 weeks require a high need for care and are stressful for patients [25]. After 2 weeks, patients were expected to be recovered enough to participate in an interview. The first author met the patients a few days before the planned interview to confirm that they still wanted to participate and had the energy to do so.

The interviews included in the study were performed by the first author between 11 and 23 days postoperatively. Five interviews took place in the participant’s care room or in a separate conversation room in the ward. Eight interviews were conducted by telephone because the participants had already been discharged from hospital before the planned interviews, or the participants did not have the energy to participate until after discharge. Interviews were digitally audio‐recorded and lasted between 10 and 49 min, with an average of 28 min. Initially, audio recordings were stored on a USB device and secured in a lockable safe at the ward. Prior to transcription, the files were transferred to the university’s data server, protected by password and two‐factor authentication. Only the designated researchers had access to the recordings and transcripts.

An interview guide was used to structure the interviews (Table 1). The interview guide was pretested with one patient who had undergone CRS combined with HIPEC. The purpose of the pretest was to assess the clarity, relevance and comprehensibility of the questions. No changes to the interview guide were deemed necessary following the pretest. The pretest interview was not included in the study sample.

**TABLE 1:** Interview guide.

Opening question:
How have you experienced your recovery up until today?

**Follow-up questions:**

Can you tell me about the first few days after the operation when you were in the ICU?
How did it feel to come to the ward?
Is there anything that has been easier or more difficult than you expected?
Can you tell me about something that has felt positive during your time on the ward?
Has anything felt less good or difficult on the ward?
Can you tell me about something that has made things easier for you during your stay? What could have been done differently to improve your wellbeing during your stay?
Is there anything else you would like to add that we haven’t talked about?

Descriptive background data were collected from the participants in connection with the interview.

### 2.6. Data Analysis

Recorded interviews were transcribed verbatim. All collected data were pseudonymised by replacing personal identifiers with sequential numerical codes. A code key was created and stored in a secure location, separate from both the audio recordings and the transcriptions. The transcriptions were then analysed using deductive content analysis, as described by Elo and Kyngäs [20]. The FoC framework was used to guide the analysis.

According to Elo and Kyngäs [20], there are three main phases (preparation, organising and reporting) in the analysing process. The *preparation* phase started with reading the transcripts several times to get a deeper understanding of the data and to get a sense of the whole. The next phase was to *organise* the text in the transcripts into predefined generic categories, which is the deductive part of the analysis. For this, an unconstrained coding matrix was developed (Table 2), for which the three dimensions in the Swedish version of FoC described by Muntlin and Jangland [22] constituted the generic categories with predefined bounds. The predefined bounds can be explained as coding rules directing the organisation of text into a suitable generic category. To be able to manage a large amount of text, text units were identified and given codes (i.e., short descriptions). Guided by the predefined bounds, text with similar codes was sorted into a suitable generic category. When all text had been organised into a generic category, it was time to finalise the analysis and describe fundamental care needs corresponding to the aim of the study. For this, the content in each generic category was inductively reviewed and synthetised.

**TABLE 2:** Generic categories with predefined bounds/coding rules based on the Fundamentals of Care framework together with subcategories/codes that emerged during analysis.

Generic category	Integrating the physical, psychosocial and relational aspects of nursing	Establishing a care partnership between nurse and patients/relatives	Considering the context in which care is provided

Predefined bounds/coding rules Include needs of	Personal hygiene and get dressed	Active listening	*Organisational level:*
	Toilet/elimination	Being empathetic	Resources
	Sleep and rest	Show compassion	Culture
	Mobility	Be committed to the patient	Leadership
	Nutrition	Being present and supportive	Evaluation and feedback
	Physical well‐being	Ensuring that goals are set	*Policy level:*
	Communication		Economy
	Being respected		Quality and safety
	Integrity		Management
	Dignity		Laws and regulations
	Emotional well‐being		Accreditation

Subcategories/codes	Loss of control	Professional nurses	High workload
	Changes in taste experiences	Feelings of loneliness	Care environment
	External influences on appetite^∗^	Feelings of safety^∗^ and be taken care of goals for the future	
	Guidelines and support		
	Environmental inhibits^∗^		
	Fatigue		
	Factors related to sleep^∗^		
	Pain management^∗^		
	Own attitude		

*Note:* Subcategories/codes marked with an asterisk (^∗^) are also related to the generic category ‘Considering the context in which care is provided’.

For the *reporting*, the Consolidated Criteria for Reporting Qualitative Research (COREQ) [26] were used.

### 2.7. Ethical Considerations

The study was carried out following the Declaration of Helsinki [27] and received approval from the Swedish Ethical Review Authority 20/01/2022 (No.: 2021‐06637‐01).

Participants voluntarily decided whether to take part in the study or not. Written consent was collected in connection with the interviews before the interviews started, and all data were handled so that no individual participant could be identified. Descriptive background variables were analysed and presented at the group level to respect the privacy of the participants [28].

### 2.8. Rigour and Reflexivity

To ensure the study’s trustworthiness, its credibility, dependability and transferability were considered during the whole process [19]. Credibility in the study was enhanced by using a coding matrix that was created before analysis began. Discussions regarding data labelling and sorting were also regularly held between the authors during the analysis process. Furthermore, the analysis process was described as clearly as possible [20]. Dependability was increased by using a semistructured interview guide. Also, one author conducted all interviews to ensure that they were held in a similar way. For transferability, the COREQ checklist [26] was followed to provide a clear description of participants, context, data collection and process of analysis, and authentic citations were used [20].

As for reflexivity, the researchers’ background and experiences could shape their interpretations [28]. The first author, a female registered nurse specialised in surgical nursing and with a 2‐year master’s degree in care science, worked at the surgical ward where participants were cared for but did not participate in their care. The other author, also a female registered nurse specialised in surgical nursing and with a PhD in medical science, did not work at the surgical ward and had no connection with the participants. Both authors have long experience of giving care to patients who have undergone CRS with HIPEC. However, to prevent the risk of bias due to the authors’ professional experience, the analysis was continuously discussed and reflected upon between the authors.

## 3. Findings

Findings are based on interviews with 13 participants. In the following, participants’ experiences of their fundamental care needs are described and presented based on the three dimensions that constitute the FoC framework (Table 2). The term ‘nurse’ includes both registered nurses and assistant nurses because the participants did not distinguish between those professions in their descriptions.

The participants were between 51 and 71 years old. Demographics are further presented in Table 3.

**TABLE 3:** Participant demographics, *n* = 13.

Variable	Frequency	Percent
Age, range (mean)	51–71 (60)	
Gender		
Men	7	(53.8)
Female	6	(46.2)
Marital status		
Living in a relationship	11	(84.6)
Not living in a relationship	2	(15.4)
Primary diagnosis		
Colorectal cancer	9	(69.2)
Appendix cancer	3	(23.1)
Pseudomyxoma peritonei	1	(7.7)
Employment		
Working	8	(61.5)
Retired	5	(38.5)

### 3.1. Integrating the Physical, Psychosocial and Relational Aspects of Nursing

The FoC framework acknowledges that physical and psychosocial care needs should be integrated with relational aspects of nursing. In the participants’ descriptions, several physical needs were closely associated with psychological needs, as well as with the relation between the nurse and patient (in the form of interaction and nursing interventions) and the context. This association was described as vital for the outcome of the participants’ care.

Some of the participants described having postoperative diarrhoea. Participants who needed help with either incontinent protection or stoma care expressed embarrassment and of losing control over a fundamental ability. This also challenged their psychological care needs of respect and dignity.

‘I had to lie there with a diaper, so you had to be turned around and stuff like that when they were going to change it, it kind of happened quite often. And you felt like *Ahh*. It became extra work for them’. (Participant 1).

However, after the nurses gave the participants information about why they had diarrhoea and what is considered normal after abdominal surgery, the participants felt relieved, and it helped them cope with altered bowel movements and personal hygiene.

Most of the participants experienced changes in taste after the surgery. Consequently, eating and drinking were described as challenges. In addition, the participants described a limited variety of hospital food, also stating that it did not taste good. More food choices were warranted, such as cold alternatives. To avoid nausea and lack of appetite, several participants described eating little and often, which promoted their ability to eat and drink.

Several participants expressed that they needed more support when trying to mobilise themselves. Also, they did not know what was expected of them and would have liked more guidelines regarding, for example, the number of steps per day or optimal time points for walks. They also felt that much of the responsibility to get up and move around was on them.

‘I have seen how cheerful they have been with older people who weren’t too happy to get out of bed. I have been able to handle that myself. But I feel that it has been entirely on me. There hasn’t been any routine, and I can sort of think that, maybe… [at] certain times, like, “At 11 after dinner, you should always go for a walk, or at that point, you should get up and go”’. (Participant 11).

A further impeding factor for movement was fear of damaging the surgical wound.

During the first postoperative days, fatigue was described as prominent, reflecting the fundamental need for sleep and rest. Both sleep and rest were hindered by factors mainly related to the care environment. The main problem was that nurses frequently interrupted the patients’ sleep and rest because they needed to check on them, control blood pressure, collect blood samples or give intravenous antibiotics. Also, anxiety and thoughts about the future made it hard to sleep. In contrast, factors that promoted sleep were having adequate pain relief and whether the nurses planned their nightshift work.

Postoperative pain was described as expected by several participants. However, almost all participants explained that they received sufficient pain relief by patient‐controlled epidural analgesia (PCEA), which not only contributed to their physical well‐being but also gave a sense of security. However, after the epidural analgesia was discontinued, pain became more difficult to handle.

Participation and information were stated as the most prominent fundamental psychosocial needs, but privacy and emotional well‐being were also pointed out. An easy‐to‐understand language by all healthcare categories was preferred, without the use of Latin terms. The participants expressed a need for more information from the surgeons, where they would take their time and sit down and explain, preferably together with relatives.

‘I had to remember, so I had questions for the next day [for the medical rounds], but then I was like, “Stop, stop, stop. Now I have a few questions.” So, I kind of had to stop them on the way out’. (Participant 9)

Another aspect that was described as warranted was information about the presence of a counsellor or psychologist and how to get in touch. The need for privacy was experienced as fulfilled when participants had single‐bed rooms, which promoted peace and quiet as well as better sleep. This need can also be correlated to the dimension ‘Considering the context in which care is provided’.

‘There are many sharing the same toilet. Like last night, I sat on the toilet for the whole evening, and I just felt, *Oh, what if someone else wants in here*? A little panic that … well, it didn’t feel good’. (Participant 12)

In relation to the need for emotional well‐being, the participants described relief at having had the surgery and being able to look forward.

### 3.2. Establishing a Care Partnership Between Nurse and Patient/Relatives

The most central aspect within the FoC framework is the nurse–patient encounter. This requires an established care partnership with a holistic view of basic care needs. Nursing interventions that establish a good care partnership are active listening, being empathetic and showing compassion, commitment to the patient, being present and supportive, and ensuring that goals are set.

Almost all participants were satisfied with the nurses’ expressions of empathy and compassion. Taking the time to sit down and listen to the participants was experienced as significant. Nurses were described as professional and positive with much patience and a caring and sweet attitude, all of which were considered important for the recovery process.

‘Everyone who works there, all their professionalism, eh, what I was worried about before was the days when you are, to be blunt, like toxic waste [due to the cytostatic]. I thought it would be very troublesome, that no one would touch me, or I wouldn′t get a hug or anything for these 5 days, but they all handled it amazingly well. I didn′t feel disgusting, or how should I say it … “dangerous”’. (Participant 4)

However, regarding commitment for the patient, some participants felt they had to ask to get the help they needed instead of the nurses proactively anticipating their needs. This led to feelings of loneliness, particularly when participants had a single‐bed room.

It was considered positive when nurses prepared the participants for what was going to happen and how, as it made them feel safe and well taken care of. Furthermore, it was perceived as important for nurses to not work solely in a task‐focused manner but instead take the time to talk and be present in the meeting, which gave participants the sense of being seen.

‘Instead of just coming in and “Now we’re going to redress the wound,” they’ve been very, uh, they′ve seen me. In that way, I felt taken care of, that they made sure that I felt well’. (Participant 6)

Participants thought that it was important to set goals for the future in order to have something to look forward to. A few of the participants had set goals during the early postoperative recovery period, but when the goals were not met, they lost their positive attitude regarding the recovery process. Some of the participants wanted more available information about how one might feel after this type of surgical procedure so they could be more prepared and able to set more realistic goals for oneself.

### 3.3. Considering the Context in Which Care Is Provided

The third dimension presents the contextual factors that affect how care can be performed. To be able to provide good care as described in the FoC framework, prerequisites are needed at both the levels of organisation and policy.

The participants wanted larger spaces, more single‐bed rooms and a more inspiring hospital environment. They described the environment on the ward as inhibiting recovery. For example, narrow corridors with walking aids and drop stands in the way unmotivated them to get up and move around. Further environmental factors that hindered the participants’ appetite were that the dining room was not a peaceful environment but rather messy and noisy. Also, noise from both the ward and the outside, as well as lamps that were illuminated at night, had a negative effect on sleep.

The participants also noticed that the nurses had many tasks to do during their shifts and did not always have the time to be more present, which resulted in the patient having to wait longer.

‘They say, “We have so much, can you wait a minute?” You sort of notice that they… Many times, when they have to go get something, they get distracted with other work along the way, so it takes maybe half an hour, an hour before they come back’. (Participant 13)

## 4. Discussion

### 4.1. Integrating the Physical, Psychosocial and Relational Aspects of Nursing

Within today’s surgical care, an early postoperative intake of food and drink is recommended to improve the resumption of bowel function [29]. However, in this study, it was found that changes in taste affected the appetite in a negative way, making it difficult to eat and drink. Furthermore, hospital food was not found appetising, with few suitable alternatives, and the dining environment was not found to be relaxing. These results are in line with earlier results reported by Muntlin Athlin et al. [30], aiming at exploring fundamental care needs in patients with a cancer diagnosis. If a more appealing and wider range of food alternatives could be offered in a suitable environment, patients might be able to improve their energy intake, which in turn promotes, for example, mobility and wound healing. Hence, eating and drinking not only is a fundamental need but could also be seen as a care strategy to enhance recovery.

The participants in this study requested a more structured plan for what was expected of them as well as support from the nurses regarding mobility. Earlier studies reporting on patients’ early phase of recovery after CRS combined with HIPEC have not described any findings related to mobility [15, 18]. However, prolonged bed rest is associated with a risk for thromboembolic complications, decreased skeletal muscle strength and pulmonary complications. A rehabilitation program can be used to reduce these risks [29], and when used in a person‐centred way, it could be a valuable tool for nurses to provide tailored support to patients and promote the establishment of a care partnership.

In the current study, the need for sleep and rest was emphasised as hindered by both environmental factors, such as noises from the ward or other patients in shared rooms and by the nurses performing routine tasks. These findings are confirmed by previous research presented by Gellerstedt et al. [31], who interviewed patients who were planned for surgery or medical treatment. It can be argued that hospitals, with their care environment, have not been designed primarily to promote recovery. Nurses may find it difficult to influence such issues; however, they can sometimes plan their care interventions so that, for example, the patients’ sleep is less interrupted.

Previous studies [15, 18] have shown that pain and pain management were experienced as troublesome during the early postoperative phase. Surprisingly, this was not a prominent finding in this current study. Instead, it was shown that most of the participants experienced sufficient pain management with the use of PCEA. This also contributed to physical well‐being and a sense of security. PCEA has been increasingly implemented during the last decade, but hospitals have different routines related to pain management, and it may be that the participants in previous studies had received different pain treatment. If so, then the use of PCEA is an effective way to promote not only the absence of postoperative pain but also physical well‐being and a sense of security. Considering the FoC framework, the issue of pain management concerns physical and psychological needs. However, it might also be related to the dimension ‘Considering the context in which care is provided’ because in Swedish healthcare, routines for pain management concern both organisational and policy levels.

Participants in this study described experiences of overwhelming fatigue, which have been reported before. As an example, Eriksson et al. [15] described that their participants expressed physical weakness as well as psychologically and physically fatigued beyond the ordinary. Due to fatigue, the participants in this current study asked for more proactive support by the nurses to help with their mobility and to remind them about food and drink. These are arguably relevant requests, given that fatigue is an expected symptom after major surgery. A proactive approach is described as important for the care relationship in the FoC framework [21]. The nurse should strive to predict and identify patients’ needs and act on them. If fundamental needs remain unmet, they will become a problem.

### 4.2. Establishing a Care Partnership Between Nurse and Patient/Relatives

Some participants in this study expressed that they had to wait to get the help or support they needed or had to ask for it. They also experienced knowledge gaps among the nurses. Despite this, participants also expressed that they were satisfied with the nurses and the care they provided. The last‐mentioned result is consistent with previous studies [15, 18]. Participants in the study by Eriksson et al. [15] described healthcare professionals as encouraging, supportive and positive. Another study by Muntlin Athlin et al. [30] found that the relation with health professionals, being either positive or negative, shaped the patients’ hospital experience. Olsson et al. [32], whose study aimed to describe the fundamental care needs and experience of nursing care in patients with small intestinal neuroendocrine tumours, showed that the patients trusted the healthcare professional and felt safe but did not receive information about what would happen during the day and why. That finding is consistent with the current study, in which the participants described the importance of receiving information about what is planned. In a study using a focused ethnographic approach observing 30 nurses who worked at three different wards in Dutch hospitals [33], the results show that several nurses had a task‐oriented way of communicating and working. Most of the observed nurses had a focus on patients’ physical needs and did not take the time to acknowledge the relational and psychosocial needs of patients. The most central part of the FoC framework is the relationship between the patient and the nurse [23]. In this current study, the participants expressed the importance of being seen by the nurse and that the nurse took the time to sit down and talk, not just work in a task‐oriented manner. The FoC framework can be seen as a comprehensive tool guiding the provision of care that requires the nurse to base their care interventions on the patient’s own experiences, abilities and wishes [23]. However, the surgical ward context itself might be a hindering factor for this. One reason was explained by Jangland et al. [34], describing that the fast‐paced culture on surgical wards had an impact on patients’ fundamental physical and psychological needs.

### 4.3. Considering the Context in Which Care Is Provided

Participants in this current study recognised that the nurses had a high workload and consequently hesitated to ask for the help they needed. However, other findings in all three dimensions pointed to several shortcomings related to the context in which care is provided. This could be considered worrying and possibly affecting both the quality and outcome of care. To provide care according to the FoC framework, nurses require sufficient job resources, both at the organisational level and at the policy level. A decisive factor is when hospital organisations prioritise and create optimal working and environmental conditions [23]. Earlier research focusing on working conditions for surgical nurses [35] showed that a consistent lack of sufficient job resources adds extra stress to an already strained work situation, which can lead to higher turnover rates and, in the worst case, that nurses choose to leave their profession.

### 4.4. Strengths and Limitations

Prior to this current study, few qualitative studies have been performed to capture the experiences of patients undergoing CRS with HIPEC, and only one of them focused on the earliest recovery period. However, that study was performed over 10 years ago, and since then, surgical interventions with subsequent care have undergone significant improvements. Hence, this study is the only current study that can provide up‐to‐date knowledge. A methodological strength is the deductive approach, for which the FoC framework was used as a guide [20]. This approach made the study theoretically grounded and helped to structure both the analysis and the reporting of results, which can increase trustworthiness and transferability [36]. In addition, the study presents a breadth of both positive and negative experiences of fundamental care needs. According to Creswell and Creswell [28], good qualitative research is characterised by the diversity of perspectives about the studied topic. However, there were also limitations, for example, that some of the interviews were relatively short because some participants did not have the energy for longer interviews. The necessary amount of data to answer the research question could vary, depending on the quality of the data and the complexity of the phenomenon. The focus of this current study could be considered as complex, as it was guided by a theoretical framework covering a wealth of aspects of care. Also, whether it was possible to obtain high‐quality data could be questioned, given that some interviews were short. One way to compensate for this potential deficiency would be to perform many interviews. However, as the data collection proceeded, the amount of new information that emerged during interviews gradually decreased. It is worth highlighting that this study was explorative, and additional research is warranted. Also, the amount of data might not necessarily be an indicator of good quality.

## 5. Clinical Implications

The findings in this study indicate that patients undergoing CRS combined with HIPEC experience a pronounced loss of control in the early postoperative phase mainly because of surgery‐related physical changes, environmental factors and uncertainties about the future. These experiences imply that the surgical procedure places great demands on patients after surgery but also that there are several shortcomings within healthcare that need to be addressed to meet the patients’ fundamental physical, psychological and relational needs. Structured, person‐centred care based on patients’ abilities, resources and fundamental needs is shown to be warranted to promote recovery.

Guided by the FoC framework, nurses should prioritise establishing a partnership with the patients and, when relevant, their relatives. With such partnership, mutual goals can be formulated, continuously evaluated, and revised in response to the patients’ changing health conditions. This includes systematic and individually tailored support regarding symptom management, provision of clear and consistent information, and promotion of the patients’ active involvement in care and decision‐making.

Nurses hold a professional responsibility to prioritise person‐centred nursing care and actively foster such partnerships, while healthcare organisations are responsible for ensuring nurses’ continuous professional development and for creating organisational opportunities for nurses to develop and work in such a manner. The FoC framework can serve as practical support for implementing and sustaining person‐centred care within clinical practice. Through an alignment between professional commitment and organisational support, high‐quality care, in which the patients’ fundamental care needs are met, can be offered to patients undergoing a demanding procedure such as CRS with HIPEC.

## 6. Conclusion

The patients in this study described mainly physical needs; however, most of those were interrelated with psychological needs and their relations with nurses. Furthermore, it was clearly described that environmental factors as well as nurses’ sometimes task‐oriented approach had a great impact on patients’ opportunities to have their physical and psychological needs met. Nursing interventions, such as providing patients with information and support, were also considered important to be able to cope with postoperative symptoms and to promote mobility. The nurse–patient partnership was described as a factor that promoted recovery, as some nurses were perceived as professional, patient and willing to sit down and talk with the patients. Factors or policies regarding the patients’ environment are perhaps difficult for the individual nurse to be able to influence. However, as this study shows, the nurse–patient partnership is an important factor in recovery, and each nurse has their own mandate to adopt a person‐centred approach. The FoC framework can be suggested as a guideline for both hospital organisations and nurses to create a high‐quality care environment.

## Funding

No funding was received for this research.

## Conflicts of Interest

The authors declare no conflicts of interest.

## Supporting Information

Additional supporting information can be found online in the Supporting Information section.

## Supporting information


**Supporting Information** COREQ‐checklist.

## Data Availability

The data that support the findings of this study are available on request from the corresponding author. The data are not publicly available due to privacy or ethical restrictions.
